# Evidence for *in vitro* and *in vivo* expression of the conserved VAR3 (type 3) *plasmodium falciparum* erythrocyte membrane protein 1

**DOI:** 10.1186/1475-2875-11-129

**Published:** 2012-04-25

**Authors:** Christian W Wang, Thomas Lavstsen, Dominique C Bengtsson, Pamela A Magistrado, Sanne S Berger, Andrea M Marquard, Michael Alifrangis, John P Lusingu, Thor G Theander, Louise Turner

**Affiliations:** 1 Centre for Medical Parasitology at Department of International Health, Immunology, and Microbiology, University of Copenhagen and at Department of Infectious Diseases, Copenhagen University Hospital (Rigshospitalet), 1014, Copenhagen, Denmark; 2 National Institute for Medical Research (NIMR), Tanga Medical Research Centre, Tanga, Tanzania

## Abstract

**Background:**

Members of the *Plasmodium falciparum* erythrocyte membrane protein 1 (PfEMP1) adhesion antigen family are major contributors to the pathogenesis of *P. falciparum* malaria infections. The PfEMP1-encoding *var* genes are among the most diverse sequences in nature, but three genes, *var1, var2csa* and *var3* are found conserved in most parasite genomes. The most severe forms of malaria disease are caused by parasites expressing a subset of antigenically conserved PfEMP1 variants. Thus the ubiquitous and conserved VAR3 PfEMP1 is of particular interest to the research field. Evidence of VAR3 expression on the infected erythrocyte surface has never been presented, and *var3* genes have been proposed to be transcribed and expressed differently from the rest of the *var* gene family members.

**Methods:**

In this study, parasites expressing VAR3 PfEMP1 were generated using anti-VAR3 antibodies and the *var* transcript and PfEMP1 expression profiles of the generated parasites were investigated. The IgG reactivity by plasma from children living in malaria-endemic Tanzania was tested to parasites and recombinant VAR3 protein. Parasites from hospitalized children were isolated and the transcript level of *var3* was investigated.

**Results:**

*Var3* is transcribed and its protein product expressed on the surface of infected erythrocytes. The VAR3-expressing parasites were better recognized by children´s IgG than a parasite line expressing a Group B *var* gene. Two in 130 children showed increased recognition of parasites expressing VAR3 and to the recombinant VAR3 protein after a malaria episode and the isolated parasites showed high levels of *var3* transcripts.

**Conclusions:**

Collectively, the presented data suggest that *var3* is transcribed and its protein product expressed on the surface of infected erythrocytes in the same manner as seen for other *var* genes both *in vitro* and *in vivo*. Only very few children exhibit seroconversion to VAR3 following a malaria episode requiring hospitalization, supporting the previous conclusion drawn from *var3* transcript analysis of parasites collected from children hospitalized with malaria, that VAR3 is not associated with severe anaemia or cerebral malaria syndromes in children.

## Background

*Plasmodium falciparum* is a major cause of mortality and disease in sub-Saharan Africa. Immunity to malaria in areas with intense transmission is acquired during childhood as a broad repertoire of specific protective antibodies to parasite-derived polymorphic variant antigens present on the infected erythrocyte surface, develops [1-3]. *Plasmodium falciparum* erythrocyte membrane protein 1 (PfEMP1) is the best characterized variant surface antigen [4-6]. A single parasite express only one or a few variants at a time [7-11] as the parasite develops from ring-shaped early forms into trophozoite stages, simultaneously with the onset of adhesion and antigenicity of the infected erythrocyte [12]. Members of the PfEMP1 family mediate the cyto-adherence of infected erythrocytes to host receptors, allowing parasites to avoid splenic clearance and an immense sequence variation within the protein family has evolved to escape counteracting host antibody responses [13,14]. The PfEMP1 family is encoded by approximately 60 *var* genes per parasite genome [6,15], which can be divided in two four groups A-E based on their 5´upstream region [16,17]. Three *var* genes, *var1* (UPSA), *var2csa* (USPE)*, and var3* (aka Type 3) (UPSA) are conserved in their full length in the global parasite population [18-22]. Apart from these, most parasite genomes contain a set of *vars* encoding more diverse domain cassettes *not* spanning the full length genes [23]. PfEMP1s are composed of multiple Duffy binding-like (DBL) and cysteine-rich inter-domain region (CIDR) domains. Among the PfEMP1s the VAR1, VAR2CSA and VAR3 variants have unique domain compositions void of CIDR domains. While VAR1 is particularly, long consisting of seven DBL domains and one CIDR, VAR3 and VAR2CSA distinguishes themselves by not containing any CIDR domains. In addition VAR3 is the smallest of the PfEMP1 proteins [15] and is the only PfEMP1 consisting of only two domains; DBLα/ζ and DBLε [23]. *Var3* genes have arisen from a recombination between a DBLζ-DBLε encoding sequence only found in the 3´end of *var* exon1 and an N-terminal DBLα1 sequence. Sequence analysis has shown that only the DBLζ-DBLε part of VAR3, which is 99% identical between VAR3 sequences, is unique to the protein sub-family [23].

VAR2CSA binds chondroitin sulphate A (CSA) in the placenta and facilitates the parasite sequestration causing pregnancy-associated malaria. Similarly, organ specific PfEMP1 mediated sequestration has been linked to severe malaria in children [24], and evidence indicates that a restricted and antigenically conserved subset of variant surface antigens causes the most severe malaria syndromes in children [25-32]. Thus, it is hypothesized that defined PfEMP1 subtypes confer specific adhesion phenotypes for the parasites [23]. Several studies have aimed to identify the *var* types expressed in severe malaria [29,30,33-36]. Most of these have relied on defining the most prevalent *var* mRNA species by PCR amplification and sequencing of a short 350 bp DBLα tag present in all *var* genes except *var2csa* and *var3*. As this approach does not capture *var3* sequences, quantitative PCR has been deployed to investigate *var3* transcript levels in patient samples [29,37]. The overall conclusion from these studies is that members of the UPSA *var* genes are associated with severe malaria in children.

Because VAR3 is conserved, belongs to group A PfEMP1 and has an unusual domain structure, this protein could play a particular important role for the parasite and in development of malaria disease. Malaria-endemic populations has been shown to have acquired antibodies that react with recombinant VAR3 protein [38], suggesting that the protein is expressed and immunogenic during natural infections. However, quantitative PCR studies of *var3* transcript levels in malaria patient samples have shown that *var3* transcripts was not associated to any particular syndrome of severe malaria [37]. A formal demonstration of VAR3 on the surface of *P. falciparum*-infected erythrocytes has never been achieved and evidence presented by Epp *et al.*[39] suggests that *var3* transcription may be regulated differently from the rest of the *var* genes and expressed in a non-mutually exclusive manner due to an atypical intron activity**.**

In an effort to unravel some of the mystery surrounding this PfEMP1, parasites of different genetic background were successfully manipulated to express VAR3 on the surface of the infected erythrocytes. Analysis of these parasites suggests that both *var3* mRNA and VAR3 protein is expressed similar to other PfEMP1s. Furthermore, in this study, evidence for *in vivo* expression of VAR3 in malaria-sick children is presented.

## Methods

### Parasite culture, *in vitro* selection and synchronization

*Plasmodium falciparum* clones 3D7 and IT/FCR3 parasites were cultured in O Rh + erythrocytes in RPMI1640 supplemented with Albumax II, as previously described [40]. Parasites were synchronized by magnet-activated cell sorting (MACS; Miltenyi BioTec) and 24 h later ring stage parasites were harvested for RNA-extraction. The developmental stage of parasites was confirmed by assessment of 500 erythrocytes on Giemsa-stained thin smears. Parasite cultures were centrifuged and 1 ml Trizol (Invitrogen) was added to the pellet to preserve RNA. 3D7 and IT/FCR3 parasites were selected as previously described [41] by repeated rounds of panning on DynaBeads coated with rabbit IgG specific for the 3D7 VAR3 double domain of PFI1820w. A more stringent synchronization of the 3D7VAR3 line was done by gelatine purification of late-stage trophozoites and schizonts and further enriched 48 h later on a MACS column. The synchronous population of parasites was cultured in six different culture flasks and harvested at six different time points of the intra-erythrocytic life cycle. The developmental stages of the 3D7VAR3 parasites at the six time points were determined by assessment of 900 erythrocytes on thin smears stained by Giemsa.

Isolate integrity of the cultures was tested using the method previously described by Snounou *et al.*[42] with the following modifications: only the second round of amplification was done using the MSP2-IC1, MSP2-FC27 and GLURP specific primer pairs. The conditions of the PCR were as follows for all three reactions: 94°C for 15 min, 35 cycles of 94°C for 1 min, 61°C for 2 min and 65°C for 2 min, followed by 58°C for 2 min and 65°C for 5 min. The PCR reaction contained: 0.125 μM of the primer pairs, 1:1 of TEMPase Hot Start DNA Polymerase (Ampliqon, VWR) and 1 μL of DNA extracted using the DNeasy Kit (Qiagen) according to the manufacturer’s instructions. A set of most common in-house used laboratory isolates (3D7, IT/FCR3, DD2, K1, and HB3) as well as *P. falciparum* negative controls, human DNA from Danish donors and blank samples, were included in the setup and the amplified PCR products were analysed by agarose gel electrophoresis.

### RNA extraction and cDNA synthesis

RNA was extracted using Trizol (Invitrogen) according to manufacturer’s instructions. Samples were treated with DNase I (Sigma) to digest any genomic DNA and tested in quantitative PCR for contamination, using a primer set, p90, for the *seryl-tRNA synthetase* gene. RNA was reverse transcribed from random hexamers, using Superscript II (Invitrogen), according to the manufacturer’s instructions (Invitrogen).

### Primer design

Quantitative primers for each *var* gene of the *P. falciparum* clone 3D7 and to the endogenous control genes *seryl-tRNA synthetase* and *fructose-bisphosphate aldolase*, have been described previously [20] with modifications [43]. Specific quantitative primers were designed for 56 *var* genes extracted from the IT/FCR3 genome available at The Wellcome Trust Sanger Institute (UK) [44] (Additional file 1). The primers were validated by testing amplification efficiency relative to p90 on 10-fold dilutions of genomic DNA using quantitative PCR. All primer pairs had amplification efficiencies >90%, and produced single band products on agarose gel electrophoresis with single melting peaks in melting curve analysis. Two cross-intron primers to detect spliced *PFI1820w*/*PFF0020c/IT4var3* (var3Xi) and *PFA0015c* (PFA0015cXi) transcripts, respectively, were designed as well (Additional file 1). The amplification efficiency of these primers was not determined; however, their correct PCR amplification product sizes from genomic DNA and cDNA of both 3D7 and IT/FCR3 parasites was confirmed on agarose gel electrophoresis. A primer pair designed to universally amplify *var3* transcripts was also used (Additional file 1). To further validate the specificity of all primers, the PCR products were sequenced, using the specific forward and reverse primer. All primer pairs were specific for their target genes.

### Quantitative PCR

Quantitative PCR was performed on a Rotorgene RG-3000 thermal cycler (Corbett Research), applying QuantiTect SYBR Green PCR Master Mix (Qiagen) with primers at 20 μM, and internal control genes *seryl-tRNA synthetase* and *fructose-bisphosphate aldolase*, used for normalization. Gene-specific standard curves were produced by determining the amplification efficiency relative to the single copy internal control gene, *seryl-tRNA synthetase*, based on quantitative measurements of 10-fold dilutions of genomic DNA and used to calculate the transcript copy number of each gene in tested cDNA. Transcript levels relative to the average level of the two internal control genes were calculated as 2^-∆Ct(*var* gene)^ and the level of internal control genes were set to 100 (User Bulletin #2: ABI Prism 7700 Sequence Detection System; Applied Biosystems)

### Protein expression and generation of antiserum

Protein expression was performed as previously described [28,46] and the rabbit antiserum raised against recombinant DBLα-DBLε double domain (~90 kDa) of the clone 3D7 PFI1820w protein from a previous study [38] was used in this present study. The antiserum was tested positive in ELISA for reactivity against the immunizing antigen [38]. Depletion of antibodies reacting with erythrocyte antigens was done by mixing equal amounts of antiserum with human O^+^ erythrocytes and incubating 24 h at 4°C. Besides the full length VAR3 protein, two full length and four multi-domain PfEMP1 proteins were included in the Luminex assay (Additional file 2). The C-terminal region of MSP3 was included as control as previously described [48].

### Flow cytometry

The surface expression of VAR3 PfEMP1 by 3D7VAR3 and FCR3VAR3 lines was assessed by flow cytometry [49]. The erythrocyte surface antigen phenotype of line FCR3VAR3 was characterized using plasma from 61 children (from age one to 19 years) living in Mgome, Tanzania (an area of holo-endemic malaria transmission), from a cross-sectional survey [50], for IgG surface-staining of infected erythrocytes. Danish plasma was used as a control. Acute and convalescent plasma from 130 under five-year-old children living in north-eastern Tanzania experiencing a malaria episode was also tested with the FCR3VAR3 line. Informed consent forms were signed by parent or legal guardians of all subjects and the trials were reviewed and approved by the Medical Research Coordinating Committee, Tanzania (NIMR/HQ/R.8a/Vol.IX/559). The data was acquired using a FC500 instrument (Beckman Coulter). Uninfected erythrocytes were analysed to exclude IgG binding to erythrocyte antigens.

### Confocal microscopy

Indirect immuno-fluorescence assays were performed on live 3D7VAR3/3D7 unselected and FCR3VAR3/IT/FCR3 unselected in order to observe the staining of individual infected erythrocytes with VAR3-antibodies. The staining was done using Alexa^TM^488 anti-rabbit IgG (Invitrogen) and DAPI and laser scanning confocal microscopy performed using a Nikon TE 2000-E confocal microscope with 60x oil immersion objective lens (DIC) as previously described [51].

### Luminex

Levels of plasma PfEMP1 domain-specific IgG were analysed as described previously [52]. In brief, lyophilized microspheres were reconstituted with distilled water immediately prior to use and diluted 1:333 in assay buffer E (ABE; 0.1% bovine serum albumin [BSA], 0.05% Tween 20, and 0.05% sodium azide in PBS, pH 7.4). Aliquots (50 μl) were dispensed into the wells of 1.2-μm filter-bottom 96-well microtitre plates (MSBVS 1210; Millipore) pre-wetted with ABE and washed three times with ABE by using a vacuum manifold (Millipore). Frozen plasma samples were thawed at room temperature, mixed by vortexing, and spun (16,000 × g for 5 min) to remove particulates. Plasma samples were diluted 1:80 in ABE and 50-μl aliquots were added to the microsphere wells. After incubation in the dark on a shaking platform (30 sec at 1,100 rpm followed by 30 min at 300 rpm), the plates were washed three times in ABE to remove unbound antibody. Biotinylated anti-human IgG (Sigma) antibody (25 μl/well at 1:500 dilution) was added to the microspheres, which were incubated and washed as described above. This was followed by use of streptavidin-phycoerythrin (Sigma) (50 μl/well at 1:500), incubation in the dark with shaking (30 sec at 1,100 rpm followed by 10 min at 300 rpm), and washing as described above. Finally, the microspheres were resuspended in 125 μl of ABE and analysed on a Luminex 100 IS instrument set to read a minimum of 100 microspheres per microsphere region. Antibody levels for each domain were expressed as median fluorescence intensities (MFI). A negative cut-off value of 249 MFI units, based on the reactivity measured in plasma from unexposed control donors was used.

## Results

### Generation of VAR3 expressing 3D7 and IT/FCR3 parasite lines

**Figure 1:**
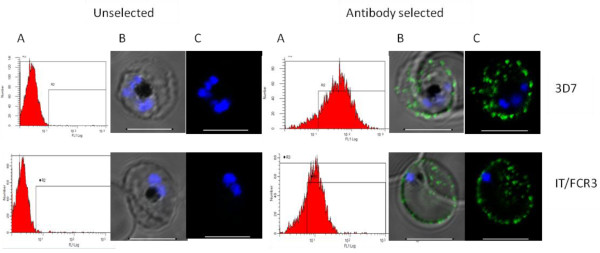
**VAR3 on the surface of*Plasmodium falciparum*infected erythrocytes.** Reactivity with anti-VAR3 antibodies of live *P. falciparum* 3D7 and IT/FCR3 before and after antibody selection with a rabbit antiserum against recombinant DBLα/ζ-DBLε of PFI1820w shown as (a) number of events/*P. falciparum*-infected erythrocytes vs. FITC flourescence intensity by flow cytometry, (b) DIC shadow-cast image with the fluorescence image superimposed and (c) the fluorescence image alone on serorepresentative infected erythrocytes in confocal microscopy. Scale bar 5 μM.

To generate *in vitro* model parasite lines expressing VAR3, parasites of two laboratory cultured parasite clones, 3D7 and IT/FCR3, were selected with rabbit antiserum against a 3D7 VAR3 (PFI1820w) recombinant protein. The expression of VAR3 on the surface of 3D7VAR3- and FCR3VAR3-infected erythrocytes, respectively, was investigated by flow cytometry and confocal microscopy using rabbit IgG specific for the clone 3D7 VAR3 PFI1820w which they had been selected with. The selected lines, 3D7VAR3 and FCR3VAR3, were recognized by the rabbit serum and stained the infected erythrocytes in a punctuate pattern typical for PfEMP1 [11,51] whereas the unselected lines were not recognized (Figure 1). Non-immune rabbit IgG did not stain the infected erythrocytes.

### VAR3 expressing parasite lines exhibit transcript patterns similar to other *var* gene expressing parasites

**Figure 2:**
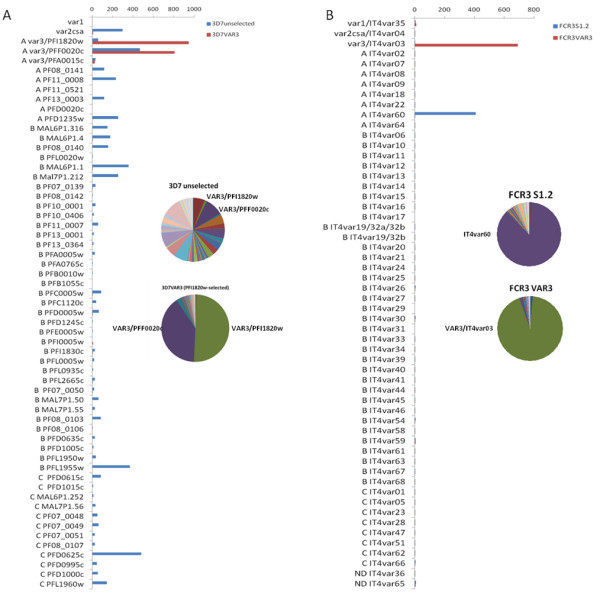
***Var*gene transcript levels in VAR3-expressing*Plasmodium falciparum*parasites.***Var* gene transcript levels relative to the average of two internal control genes before and after antibody selection of *P. falciparum* (a) 3D7 and (b) IT/FCR3 parasites, using Dynabeads coated with rabbit IgG specific for VAR3 PfEMP1. The average of control gene transcript levels is set to 100. The distribution of *var* transcripts before and after selection is also shown (circular diagrams). Transcript levels were measured by quantitative PCR using a set of primers that amplify 59 and 56 *var* genes from clone 3D7 and IT/FCR3, respectively. A cross-intron primer specific for the clone 3D7 *var3* genes, *PFI1820w* and *PFF0020c*, and the clone IT/FCR3 *var3* gene, *IT4var03*, showed similar transcript level as the corresponding exon 1 primer pairs (Table 1). The grouping of *var* genes are as previously described [16,17,23].

**Figure 3:**
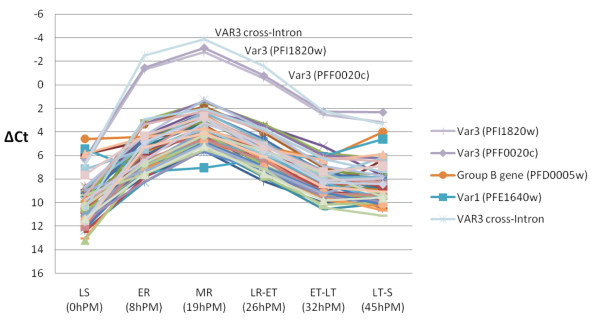
**Transcript levels of 59*var*genes during the intra-erythrocytic life cycle of parasite line 3D7VAR3.** Transcript levels were measured by quantitative PCR and shown as amplification efficiency corrected ∆Ct-values relative to internal control genes. Annotations of the most prominent transcript levels are shown. LS: Late stages; ER: Early Ring; MR: Mid Ring; LR: Late Ring; ET: Early Trophozoite; LT: Late Trophozoite; S: Schizont. hPM: h post magnet-activated cell sorting.

The *var* transcript profiles of the selected 3D7VAR3 and FCR3VAR3 and unselected 3D7 and IT/FCR3 parasite lines were investigated using *var* gene specific primers in quantitative PCR. The transcript levels of all *var* genes relative to internal control genes are shown in Figure 2. The selected lines, 3D7VAR3 and FCR3VAR3, showed markedly higher levels of *var3* transcripts, *PFI1820w* and *IT4var3*, than the unselected lines and the *var3* transcript levels were similar to those observed for *var* genes *in vivo*[53] and other adhesion-selected *in-vitro* parasite cultures with verified erythrocyte surface expression of PfEMP1 [20,28]. Interestingly, antibody selection of 3D7, which harbour three *var3* genes, resulted in selection of parasites expressing two of these genes (*PFI1820w* and *PFF0020c*) but not parasites expressing the third (*PFA0015c*) (Figure 2a). A specific cross-intron primer pair confirmed the correct splicing of *var3* transcripts (Table 1). To investigate whether the pattern of *var3* transcription diverge from that of other *var* genes, the 3D7VAR3 line was synchronized and RNA isolated throughout the intra-erythrocytic asexual lifecycle. The two *var3* genes, *PFI1820w* and *PFF0020c*, were the dominant *var* transcripts during the entire cycle with the exception at 0 h post MACS (PM), with a peak in transcript level at 19 h PM corresponding to mid ring stages (Figure 3) and thus exhibits a transcription pattern similar to *var2csa* in a VAR2CSA-expressing parasite line [43] and four group A *var* genes in the 3D7-Lib parasite line [45].

**Table 1:** **The RNA levels of*var3*in patient A and B parasite isolates and four laboratory parasite lines**

Sample Primer pair	**Patient A**	**Patient B**	**3D7 VAR3**	**FCR3 VAR3**	**FCR3 IT4var60**	**FCR3 IT4var59**
**DBLε8 (var3Global)**	187.6	419.9	1837.9	701.3	0.9	0.3
**PFI1820w (3D7)**	ND	ND	1055.6	ND	ND	ND
**PFF0020w (3D7)**	ND	ND	807.8	ND	ND	ND
**PFA0015c (3D7)**	ND	ND	17.7	ND	ND	ND
**IT4var3 (FCR3)**	ND	ND	ND	696.4	0.3	0.1
**VAR3Xi (3D7 and FCR3)**	ND	ND	1492.9	621.2	0.7	0.0
**PFA0015cXi (3D7)**	ND	ND	17.7	0.0	0.0	0.0

RNA level is shown relatively to an average RNA level of the housekeeping genes, *seryl-tRNA synthetase* and *fructose-bisphosphate aldolase*, set to 100.

ND: Not done.

### Serorecognition of 61 plasma samples from Mgome of FCR3VAR3

**Figure 4:**
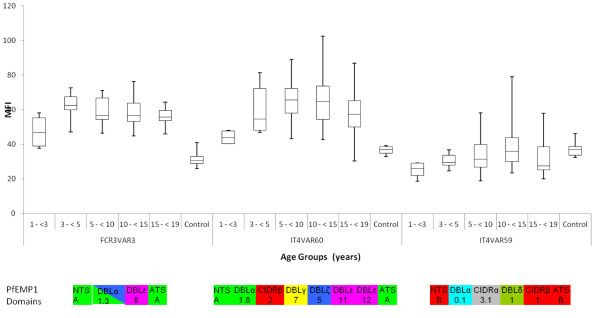
**Immune serum reactivity to three IT/FCR3*Plasmodium falciparum*parasite lines.** The reactivity of 61 plasma samples from children living in malaria endemic Mgome, Tanzania, against three parasite lines expressing VAR3 (Group A PfEMP1), IT4var60 (Group A PfEMP1) and IT4var59 (Group B PfEMP1), respectively, was measured by flow cytometry and divided into age groups: **[**1- <3] n = 4; [3- <5] n = 6; [5- <10] n = 23; [10- <15] n = 19; [15- <19] n = 9. Danish controls n = 8. The PfEMP1 domain structures as previously described [23] are shown below each parasite seroprofile. Ends of whiskers in the boxplots represent the smallest and largest median of FITC fluorescence intensity (MFI) values in the given age group.

The IgG reactivity to live FCR3VAR3 parasites in plasma from 61 individuals between one and 19 years of age living in a high transmission village of Mgome, Muheza Tanzania, was evaluated by flow cytometry (Figure 4). The 61 individuals were split into five different age groups (one to three, three to five, five to 10, 10 to 15, 15 to 19) and eight Danish donors were used as controls. The level of reactivity was compared to that of parasites expressing a Group A *var* gene, *IT4var60*, thought to mediate rosetting [54] and parasites expressing a Group B *var* gene, *IT4var59* (Additional file 3). The domain structures of the three expressed PfEMP1 proteins are shown as well (Figure 4). The FCR3VAR3 parasites were recognized by similar levels of IgG in children’s plasma as the parasites expressing *IT4var60* and by higher levels of IgG than the parasites expressing group B *IT4var59* (Figure 4). For the *var3* and *IT4var60* expressing parasite lines the <3 year olds showed the lowest IgG level but the level rose to a plateau already at the age of 3 to <5 years old. All 61 children had higher IgG levels against these two parasite lines than the Danish control donors. In contrast, the *IT4var59* parasite line was only recognized by a few of the Mgome plasma donors with a peak in the group of 10 to 15 year olds.

### Acute-convalescent plasma pairs and patient parasite isolates

**Figure 5:**
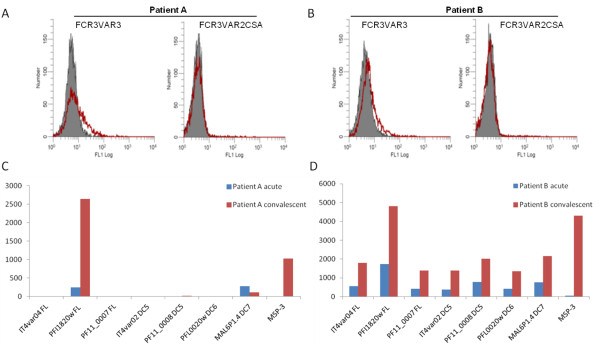
**Reactivity to VAR3-expressing*Plasmodium falciparum*parasites after a malaria episode.** The recognition of two *P. falciparum* parasite lines IT/FCR3 expressing VAR3 and VAR2CSA (a, b) and recombinant proteins (c, d) with plasma from patient A (a, c) and B (b, d) during a malaria episode (acute, grey shades) and after recovery (convalescent, red lines). The reactivity is shown as number of events/*P. falciparum*-infected erythrocytes vs. FITC flourescence intensity by flow cytometry. The acquisition of IgG to seven multi-domain and full length (FL) PfEMP1 proteins and MSP3 antigen of *P. falciparum* was measured by bead-based Luminex technology as previously described [48]. Results are expressed as median fluorescent intensity (MFI) subtracted the reactivity of unexposed control donors. The PfEMP1 domains representing domain cassette (DC) 5, 6 and 7 are as defined by Rask *et al.*[23].

The IgG reactivity to the FCR3VAR3 parasite line and recombinant VAR3 protein was investigated in acute and convalescent plasma from 130 children under five years of age from high malaria transmission villages of Korogwe, Tanzania, experiencing a malaria episode. Antibodies to FCR3VAR3 was observed in 15 (11%) of the acute patient plasma samples. Only two boys, patient A and B, appeared to acquire IgG to VAR3 but not to VAR2CSA expressing parasites after being sick and hospitalized with malaria (Figure 5a and b). An increased level of VAR3 IgG in patient A and B convalescent plasma was also found by luminex assay assessment of IgG levels to recombinant full length VAR3 and a panel of six different multi-domain and full length PfEMP1 proteins (Figure 5c and d). Patient A and B were hospitalized in north-eastern Tanzania at the age of 2^2^/_3_ and 5 years old with body temperatures of 39.5°C and 39.8°C, parasitaemias of 266,735 and 159,470 parasites/μL, Blantyre Coma Scores of 5, lactate levels of 3 mM, and haemoglobin values of 8.6 and 10.8 g/dl, respectively. Patient B appeared to have a broader anti-PfEMP1 IgG repertoire than patient A, which could be due to a difference in exposure as patient B is almost twice the age of patient A. Total RNA from the infecting parasites was isolated and the level of *var3* transcripts was investigated using universal and 3D7 and IT/FCR3 *var3* specific primers in quantitative PCR. The *var3* transcript levels in these samples were lower than in the VAR3-selected laboratory parasite lines (Table 1), but at levels previously considered high in *var* transcript analyses of field samples (Additional file 4). The transcript levels of other *var* genes in the samples were assessed using a set of 42 *var* subtyping quantitative PCR primers [37] (Additional file 4). These data indicates that apart from *var3*, other group A and DC8 encoding *var* genes are expressed at high levels in the two patients.

## Discussion

The PfEMP1 mediated parasite sequestration causes harmful inflammation and severe pathology of malaria disease and evidence suggests that particular PfEMP1 subtypes are associated with different syndromes of malaria (reviewed in [55]). In pregnant women, malaria is caused by VAR2CSA mediated parasite sequestration in the placenta [53] and the subgroup of UPSA flanked *var* genes which includes the conserved *var3* variant has been associated with severe malaria in children [28-32]. The particular involvement of VAR2CSA in pregnancy malaria and the ubiquitous and conserved nature of VAR2CSA make it an apparent vaccine target. The same might apply for VAR3, but no particular function has been ascribed to VAR3 and analysis of *var3* transcripts in patient isolates [29,37] does not predict a particular association with severe malarial disease in children. Nevertheless, the data presented in this paper indicate that VAR3 expressing parasites are viable *in vivo* and can bestow significant proportions of parasite populations in children sick from malaria. Specifically, IgG from children down to the age of one year reacted with VAR3 PfEMP1-expressing parasites where the level of recognition seemed to increase with age and find a plateau within the first three years of age. The VAR3-expressing parasites were better recognized by the children´s IgG than a parasite line expressing a Group B *var* gene. Although VAR3 is less polymorphic than Group B PfEMP1s, these data may indicate an earlier exposure to VAR3-expressing parasites than to Group B PfEMP1-expressing parasites, suggesting a particular involvement in early childhood malaria as predicted for the UPSA type PfEMP1 in general [17,28-30]. Of the 130 acute-convalescent samples from children under five years of age, only two children were found to acquire IgG antibodies to VAR3 as a result of an acute malaria episode requiring hospitalization. Patient A suffered from hyperparasitaemia but neither of the two patients was anaemic nor showed signs of cerebral malaria. Parasites isolated at hospitalization were found to transcribe *var3*-like genes at high levels, which together with the acquired antibodies indicate that VAR3-expressing parasites have contributed to the development of malaria symptoms in these patients. However, other *var* type transcripts were also detected in these patients and thus the VAR3-expressing parasites did not appear to be the only cause of disease in these two patients.

The pattern of Tanzanian children´s acquisition of antibodies to live VAR3-expressing parasites compared to UPSA and UPSB type PfEMP1-expressing parasites indicate a virulence of VAR3 similar to UPSA type PfEMP1. Yet, in spite of the conserved nature of *var3* and the presence in almost every parasite genome, it is striking that only a fifth of 1,342 individuals from malaria-endemic areas in Tanzania showed acquisition of antibodies to this PfEMP1 [38]. This may reflect either that VAR3 is not very immunogenic and/or parasites expressing VAR3 are not very virulent as also suggested by QPCR studies [29,37] and the acute/convalescent plasma study described here.

These interpretations of *var3* transcribing parasites and VAR3 antibodies in malaria patients can be challenged if *var3* genes are regulated and expressed abnormally as appears to be the case for *var1.* Studies of laboratory parasite lines show that *var1* is constitutively transcribed throughout the blood stages cycle, while the transcription of other *var* genes is silenced when parasites develop into schizonts [56]. In spite of this and the fact that malaria endemic populations develop anti-VAR1 antibodies [38], VAR1 erythrocyte surface expression has not been demonstrated. Analysis of available sequenced parasite genome shows that *var1* often lacks or has a truncated exon2 which may cause a corrupted transcription regulation resulting in protein synthesis and host antibody development upon parasite destruction. It remains unclear if VAR1 serves a particular function for the parasite and why *var1* is maintained in the population. Indeed, Epp and colleagues [39] found that the atypical *var* intron of the *PFF0020c var3* gene did not show any transcriptional activity as opposed to other non-VAR3 *var* introns. Non-coding RNA is transcribed from *var* introns and is physically associated with chromatin and believed to be involved in the transcriptional regulation of *var* genes [39]. Thus, it was speculated that *var3* genes in general could be regulated differently from other *var* genes. From the present data it does not seem that *var3* genes are transcribed and expressed differently from other *var* genes. In both 3D7VAR3 and FCR3VAR3 *var* genes other than *var3* were silenced and the *var3* transcript profile during the intra-erythrocytic life cycle was similar to that of other *var* genes in other parasite lines [43,52] and predicts as expected the surface expression of VAR3 in early trophozoite stages.

Also the VAR3 expression on the erythrocyte surface appeared similar to that of other PfEMP1. Antibodies raised towards a recombinant 3D7 VAR3 variant successfully selected VAR3 erythrocyte surface-expressing parasites from both 3D7 and IT/FCR3 parasite populations. The surface expression of VAR3 was confirmed by antibody staining in both flow cytometry and confocal microscopy with expression levels and a punctuate pattern characteristic for PfEMP1 expression in knobs. While the VAR3 in the IT/FCR3 line most likely is the only PfEMP1 on the infected erythrocyte surface, it is possible that both PFI1820w and PFF0020c VAR3 PfEMP1s are expressed by the same parasites, as previously seen in 3D7 parasites [11].

## Conclusions

In conclusion, *var3* is transcribed and the encoded protein expressed on the surface of infected erythrocytes in the same manner as seen for other viable *var* genes. VAR3-expressing parasites sustain *P. falciparum* infections in children living in malaria-endemic areas although only found in a fraction of malaria hospitalizations and not associated with severe anaemia or cerebral malaria syndromes. Whether this structurally unique and conserved PfEMP1 confer parasites expressing it with a rare functional specialization remains unclear, but further studies of the cyto-adherence phenotype of the established VAR3-expressing parasite lines may elucidate this.

## Competing interests

The authors declare that they have no competing interests.

## Authors' contributions

CWW carried out molecular biology studies, analysed data and wrote the paper. TL and TGT participated in the design, coordination and analysis of the study and helped to draft the manuscript. LT designed and carried out molecular biology studies, analysed data and helped to draft the manuscript. DCB, SBS, AMM and MA carried out molecular biology studies and revised the manuscript. PAM and JPL participated in the coordination of the study and revised the manuscript. All authors read and approved the final manuscript.

## Supplementary Material

Additional file 1 **Specific*var*gene primers used for Q-RT PCR.** Click here for file

Additional file 2 **Primers used for*Plasmodium falciparum*erythrocyte protein 1 full length (FL) and multi-domain expression.** Click here for file

Additional file 3 ***Var*gene transcript levels of*Plasmodium falciparum*IT4var59 parasites.***Var* gene transcript levels of IT4var59 parasites relative to the average of two internal control genes. The average of control gene transcript levels is set to 100. The distribution of *var* transcripts is also shown (circular diagram). Transcript levels were measured by quantitative PCR using a set of primers that amplify 56 *var* genes from clone IT/FCR3. The grouping of *var* genes are as previously described [16,17,23]. Click here for file

Additional file 4 **Transcript levels of*var3*in patient parasite isolates**. Transcript levels of *var3*-DBLε8 in *P. falciparum* parasites isolated from 130 hospitalized children (little box; red dots: patient A and B) and the *var* subtype transcript levels in patient parasite isolates, A and B. The levels are relative to the average of two internal control genes, which are normalized to 100. Transcript levels were measured by quantitative PCR using a set of 42 subtyping quantitative primers [37]. Click here for file
