# Supplementary material for: Evidence for in vitro and in vivo expression of the conserved VAR3 (type 3) plasmodium falciparum erythrocyte membrane protein 1
Source: Malar J. 2012 Apr 25;11:129. doi: 10.1186/1475-2875-11-129 (PMC3407477; doi:10.1186/1475-2875-11-129)
Supplement: Additional file 1 — Specificvargene primers used for Q-RT PCR. [file 1475-2875-11-129-S1.pdf]

Additional file 1 Specific *var* gene primers used for Q-RT PCR.

Target gene	Forward primer	Reverse primer
IT4var01	TGCAATGTAACACACTCACG	CACTATACCACAGGCATCTTC
IT4var02*	GTGCCACTACAGCTTATAGTACC	GCATATTCTTTGTCGTTGTCTTTCC
IT4var03*	ACATAGTGGATTGACAGGTGGTG	GGCTTTCCATACTTGATCTCTGTTCG
IT4var04	CACGACATTAACAATACATGCAGA	CATTGCATTCACAGACATTGG
IT4var05	GGTAGTGACGAAACTGTTGG	ACTTAAACTTTCGCACGGATAC
IT4var06	GTAACATATCCAAAGGATGAACGTCG	CCCATCTATCTTTACAAGGATGCTCC
IT4var07*	CATCCTTGCGCTGGTAGAAACAAAAC	ATTGCCTCCTGCGATAAGGAGCAC
IT4var08*	CGAATCGTAATACTAGTCGTTCAAG	GGAGGCATATAAGCTTCATTCTCTG
IT4var09	GCAAGGGCAATTTGGATGATG	GGAGTCGAGATTACGTTTGTTAC
IT4var10	GGTATCCGTGCAAAGAGTTAAGTAG	GCACATGCTCCTATACCATCCC
IT4var11	CAATGAAAGTCGTAGTGCTCCTAAC	GCTACTCTGTGATGTTCTATCATTCC
IT4var12/AAB06961	CAAACGATCATTCCAATGCTAAACG	CCGCAGGTTTCCATCCATCTTC
IT4var13	TCGGGCAACAACACTATCAA	CCCCATTCAATAAACCATCG
IT4var14	AAACCGACACAACAACCGACGACGAC	ACTATTTCGCACGCATCTGGTGGC
IT4var15	TGGTGAAGCCGCCATGTGACATAG	TAGTTGGTATGCAACGCCAGCCGTAG
IT4var16	ACCGGAAGCACCACAAGAAC	GCACCACTTATGCATTTCCATCC
IT4var17	ACATTTGTCCGCAAACATCA	AGGTGTTGTCCGTGTTCTCC
IT4var18*	GCAAGAAGGTGAAGGATCATATAGG	GCCATCATGACCCTCCTTAAAC
IT4var19/32a/32b	TGGTGGTAACACGGAGTCAA	AAGGTTTGGGGGTTTTGTTC
IT4var19/32b	TGTGACTGACTACAGTGATGCCAAAC	TGATGCTTCTGACAAACGTCCTTGC
IT4var20	ACCAACAAGACTGCCAAGGA	CCATGAAATTTTGCTTGTGA
IT4var21	TTGAAAATCCGGAGAATCTTTG	GGCTGGTACCACTATTTTCGTC
IT4var22	CGTAAATACCCTTATGACCATGC	GGGTTACAATGATCTATTGCCTG
IT4var23	ATCAACACCACCAAGACCTCGACCAC	CTGTAAACGCAATGCCAACACTCCAG
IT4var24	AAATCTGAATGCAGCGTGTGACC	TTCAACTCCACCCTTACCTGCCTC
IT4var25	AAAGACGGCACTACTCTCCAAGCAGC	TCGTCTCCTTGGTGGCACACATATGG
IT4var26	GAGCACGAGGTAAGTTGCGTTATG	CTAGCACCACCACCTGATATTTCTG
IT4var27	CTGCAGGTGGGGATGATATT	AACGTCCTTGCAAGCCTTTA
IT4var28	TTGAGTGGATAGATAAGCAACG	CATAATTAGTAGCAGTTTCTCCACC
IT4var29	GCGAATGGGAACCTAATGAC	CGGTTACACCAGCTTCTGCT
IT4var30	GAAGAGACGGTCGCAAAGAC	TTGGTTGGACAGGCATTTTT
IT4var31	CAAGATGGCAGCATTGAAAA	CGCCTCCTTCTGCATCTTAC
IT4var33	GTACGACGAAGTGAAGAATGGTGAGG	TAATCAAGTCCGCACGGATGAAGGC
IT4var34	CAACAACAGAGAAGTCAGTAGAC	CACTACCACTTGATACACATTTCC
IT4var35	CAACAACAGAATCTGACGTGGGC	TTTGGATTACAACCCTCTATTCCACC
IT4var36	ACGTGTTCTGTTTCGTGTCGTCC	GCAGCTCGTCTTAGCCTCCTAATACC
IT4var39	CATACTGCCAAGCATGTCCA	CGACTTTTCTTCCTTCTTTGC
IT4var40	GGTGGTAGTACACAGGATGAAGATG	TCACTTTACCCAAATCTCCTTGC
IT4var41	GGACATGTCAGGTCATCACG	ACCATTCTGCCCATTCAGTC
IT4var44	TGAAAGGTATCCGTGTACAGAG	CACCATTCTTACGATCATTACCTTC
IT4var45	TTACCGCCTTCGTGGAAAGTGCTG	TTGTAGTCCTCCCGCTCCTTCTTGTG
IT4var46	CACCAGTAAATGTGTGCGATATAG	GATACATATGGCACCCTTATCAC
IT4var47	TGAAAGGAAAGTTGTCACTAGCAAG	CCACATGGCTTCGTATTACCCTTAG
IT4var51	TTGGAAGGAAAGTTGTCACTAGCACC	CGAGCAGCAAGTTTCTCACCTTTATC

IT4var54	CCTTACGTTGAGTGGATAGATAACC	TCAGTACTAATACTACGTGCTCTTC
IT4var58	GTGGAGGTTTGCAGCATAGTGGACAG	TTTCCAGTGGCTTCACCCGTGTTACC
IT4var59	GGTCACACCAATGATCCATGC	CACATTCACCGCCACTTTCTTTAG
IT4var60	CCCTTGTAAAAGTGAACTCACCA	CGAGGGGTTTTTCGGATTAT
IT4var61	ACCGCAGAAGACACCACAGAAG	GGTTGCACGCGGCATTCAAATTATTG
IT4var62	GCCATGTGAAATAGTGGCTGAAC	GGCTTCACCACTTGATACACATTTCC
IT4var63	AAAGGATACGACATCACTGGAC	GCCATTTACATATTTGGTTGGACAC
IT4var64	GAAATCCTTGCGATGGAAGAAATC	TGTCGTCTTCTTGGTGGAGC
IT4var65	CAACAGCAACACCGAAAGAA	ACTTCCTCCTTGGCTTTGGT
IT4var66	GTGTTCTCTCAAATATGGACCAG	GACTTTCACTCTCACTACTTGTG
IT4var67	CAGCTCTATCACGTAGTTGGAGTG	GATTAAGCTGGCATGGATCTTCTG
IT4var68	GAGCGATTGGTAAATTACGTTATGG	CACCACCACCACCTGATATTTC
Var3Xi	CTTAATATCTACGAAGACCCCAACA	GTAATATCCGTATCATATCTACTTGACG
PFA0015cXi	CTTAATATCTACGAAGACCCCAACA	GTAATATCCGTATCATATCTACTTCATG
DBLα1.3var3Global	CGACGTAATCATAGACGTAGAGGTT	CAAGGGACTCAACTTTACACTCATT
DBLε8var3Global	AAGAGGATCTACTTAATGCTGCTTTTAG	AACTGAACTTCATAGCCTCATATGC

*Previously described [45].
