# Supplementary material for: Evidence for in vitro and in vivo expression of the conserved VAR3 (type 3) plasmodium falciparum erythrocyte membrane protein 1
Source: Malar J. 2012 Apr 25;11:129. doi: 10.1186/1475-2875-11-129 (PMC3407477; doi:10.1186/1475-2875-11-129)
Supplement: Additional file 2 — Primers used forPlasmodium falciparumerythrocyte protein 1 full length (FL) and multi-domain expression. [file 1475-2875-11-129-S2.pdf]

Additional file 2 Primers used for *Plasmodium falciparum* erythrocyte protein 1 full length (FL) and multi-domain expression.

Protein name	PfEMP1	Genome	Domains							Group	Forwardprimer	Reverseprimer
var2csa FL	IT4var04	FCR3	DBLpam1	DBLpam2	CIDRpam	DBLpam3	DBLepam4	DBLepam5	DBLe10	E	a	a
30/28-2 FL	PFI1820w	3D7	DBLa13	DBLe8						A	b	b
420/421 FL	PF11_0007	3D7	DBLa0.15	CIDRa3.2	DBLd1	CIDRb1				B	CGGATCCCATGGAGCCGCATGGAGGTAG	CTGCGGCCGCTACAAGCGCCTTTTTCCTCTGC
238/245	IT4var02	FCR3	DBLg12	DBLd5	CIDRb3	DBLb9				A	CGGATCCCTGCGAAATAGTGGAGGATC	TGCGGCCGCTACACTTACACGCACCCTTATAC
232/235	PF11_0008	3D7	DBLd5	CIDRb4						A	CGGATCCCTGCGAAATCGTGGATAAAAC	TGCGGCCGCTACAAAAAGTGGGTGCAACG
51/56	PFL0020w	3D7	DBLg14	DBLz5	DBLe4					B	CTCTAGAATGTAATCAGAAAAGTGACGC	TGCGGCCGCTGTATTTTTCTATTATTTCATCAAAATTAC
69/74	MAL6P1.4	3D7	DBLe2	DBLe7	DBLe3					B	CGGATCCCTGTGGAAATTTTAGAACTC	TGCGGCCGCTATATTCAGAGAGACAATGAAC

a Previously published [47]

b Previously published [48]
