# Supplementary figures and images for: Evidence for in vitro and in vivo expression of the conserved VAR3 (type 3) plasmodium falciparum erythrocyte membrane protein 1
Source: Malar J. 2012 Apr 25;11:129. doi: 10.1186/1475-2875-11-129 (PMC3407477; doi:10.1186/1475-2875-11-129)

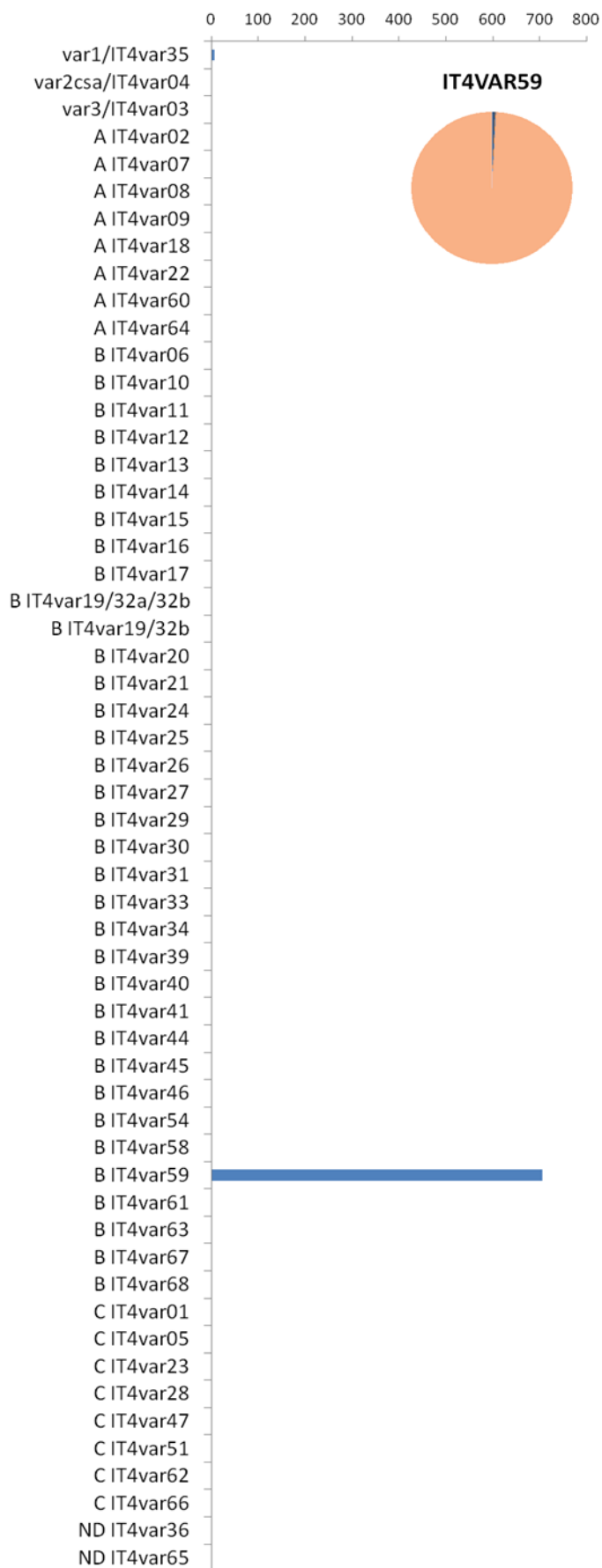

Supplement: Additional file 3 — Vargene transcript levels ofPlasmodium falciparumIT4var59 parasites.Var gene transcript levels of IT4var59 parasites relative to the average of two internal control genes. The average of control gene transcript levels is set to 100. The distribution of var transcripts is also shown (circular diagram). Transcript levels were measured by quantitative PCR using a set of primers that amplify 56 var genes from clone IT/FCR3. The grouping of var genes are as previously described [16,17,23]. [file 1475-2875-11-129-S3.pdf]

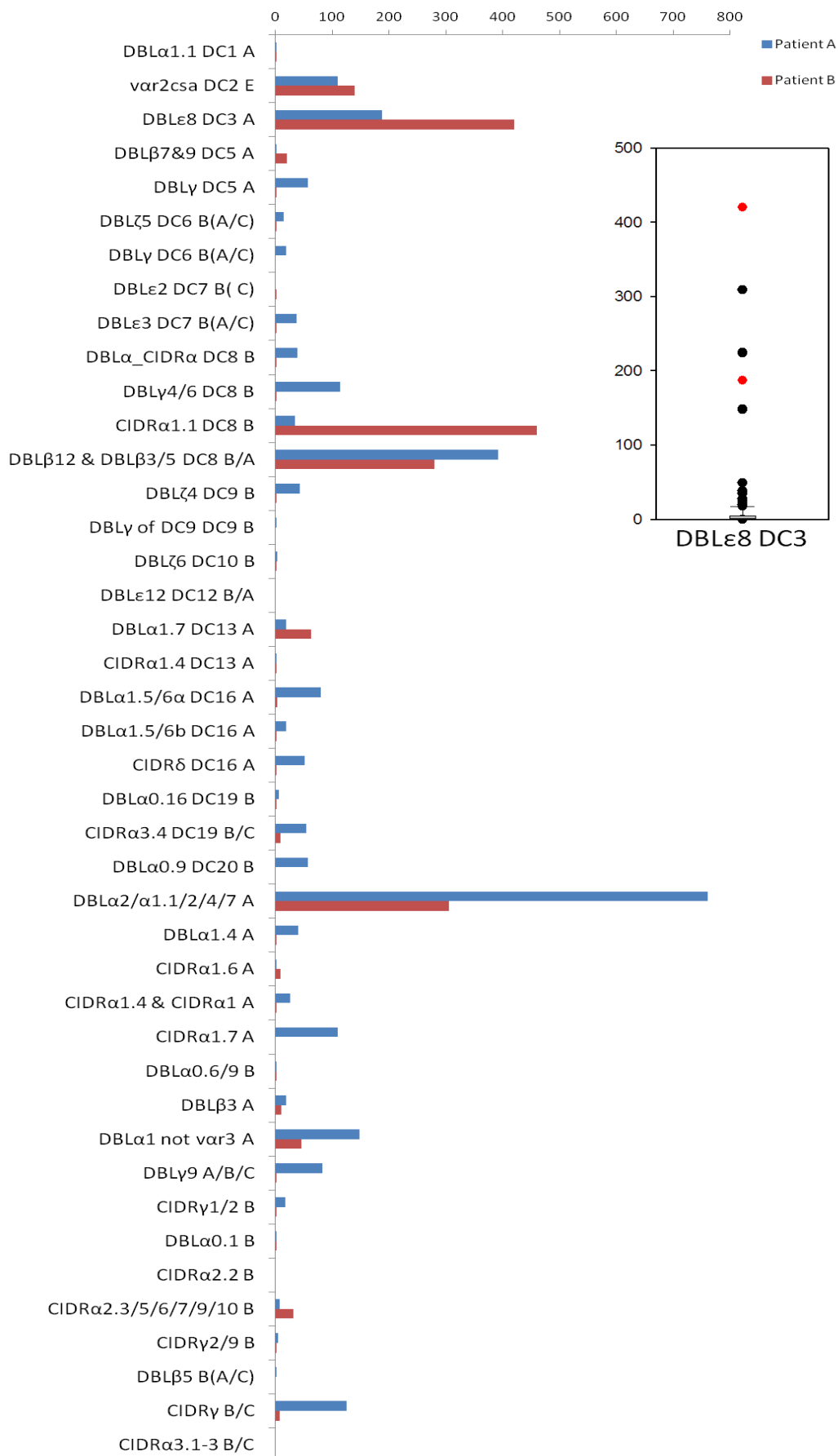

Supplement: Additional file 4 — Transcript levels ofvar3in patient parasite isolates. Transcript levels of var3-DBLε8 in P. falciparum parasites isolated from 130 hospitalized children (little box; red dots: patient A and B) and the var subtype transcript levels in patient parasite isolates, A and B. The levels are relative to the average of two internal control genes, which are normalized to 100. Transcript levels were measured by quantitative PCR using a set of 42 subtyping quantitative primers [37]. [file 1475-2875-11-129-S4.pdf]
